# Fabrication and Biological Activities of All-in-One Composite Nanoemulsion Based on *Blumea balsamifera* Oil-Tea Tree Oil

**DOI:** 10.3390/molecules28155889

**Published:** 2023-08-05

**Authors:** Yue Zhu, Teng Chen, Tingting Feng, Jiaojiao Zhang, Zejing Meng, Ning Zhang, Gang Luo, Zuhua Wang, Yuxin Pang, Ying Zhou

**Affiliations:** 1 College of Pharmaceutical Sciences, Guizhou University of Traditional Chinese Meidicine, Guiyang 550025, China; 2 Nano-Drug Technology Research Center, Guizhou University of Traditional Chinese Medicine, Guiyang 550025, China; 3 College of Food and Health, Zhejiang A&F University, Hangzhou 311300, China; 4 School of Acupuncture-Moxibustion and Tuina, Guizhou University of Traditional Chinese Medicine, Guiyang 550025, China; 5 Key Laboratory of Medical Microbiology and Parasitology, Key Laboratory of Environmental Pollution Monitoringand Disease Control, Ministry of Education, School of Basic Medical Sciences, Guizhou Medical University, Guiyang 550025, China

**Keywords:** nanoemulsion, *Blumea balsamifera* oil, tea tree oil, glycyrrhizic acid, anti-inflammatory, antibacterial

## Abstract

Nanoemulsion is a new multi-component drug delivery system; the selection of different oil phases can give it special physiological activity, and play the role of “medicine and pharmaceutical excipients all-in-one”. In this paper, we used glycyrrhizic acid as the natural surfactant, and *Blumea balsamifera* oil (BB) and tea tree oil (TTO) as the mixed oil phase, to obtain a new green functional composite nanoemulsion. Using the average particle size and polydispersion index (PDI) as the evaluation criteria, the effects of the oil ratio, oil content, glycyrrhizic acid concentration, and ultrasonic time on the nanoemulsion were systematically investigated. The stability and physicochemical properties and biological activities of BB-TTO NEs prepared via the optimum formulation were characterized. The optimal prescription was BB: TTO = 1:1, 5% oil phase, 0.7% glycyrrhizic acid, and 5 min ultrasonication time. The mean particle size, PDI, and zeta potential were 160.01 nm, 0.125, and −50.94 mV, respectively. The nanoemulsion showed non-significant changes in stability after centrifugation, dilution, and 120 days storage. These nanoemulsions were found to exhibit potential antibacterial and anti-inflammatory activities. The minimal inhibitory concentration (MIC) of BB-TTO NEs against *Escherichia coli*, *Staphylococcus aureus*, and *Pseudomonas aeruginosa* is 2975 μg/mL, 2975 μg/mL, and 5950 μg/mL, respectively. A lower level of inflammatory cell infiltration and proportion of fibrosis were found in the synovial tissue of AIA rats treated with BB-TTO NEs. These findings demonstrate that the BB-TTO NEs produced in this study have significant potential for usage in antibacterial and anti-inflammatory areas.

## 1. Introduction

A nanoemulsion (NE) is a thermodynamically stable system consisting of an oil phase, surfactant, cosurfactant, and aqueous phase, and can be divided into oil-in-water NE (O/W), water-in-oil NE (W/O) and bi-continuous NE (B.C) [1,2,3]. As a sophisticated and efficient encapsulation, stabilization, and controlled release delivery system for active substances (drugs), NE exhibits a remarkable capacity to enhance the stability, solubility, dispersion, and bioavailability of its constituents, through precise manipulation of the oil and water phases’ composition [4,5,6], the surfactant varieties, and the emulsification conditions. As such, NEs have been widely used in medicine [7,8], food [9], cosmetics [1], and other fields.

Essential oils (EOs) are complex blends of aromatic metabolites extracted from different plant parts, including leaves, bark, flowers, and roots, and can be divided into a volatile and non-volatile portion. Volatile compounds represent 90–95% of Eos, including monoterpenes, sesquiterpene hydrocarbons, and their oxygenated derivatives, aldehydes, alcohols, and esters. The non-volatile portion (5–10% of the whole EO) comprises hydrocarbons, fatty acids, sterols, carotenoids, waxes, cumarines, and flavonoids [10]. *Blumea balsamifera* oil (BB) was extracted and isolated from the leaves of *Blumea balsamifera (*L.*) DC*. [11], which has broad spectrum antibacterial [12], anti-inflammatory [13], antioxidant [14], and other biological activities. Tea tree oil (TTO) is a colorless-to-light-yellow oily liquid with an aromatic smell, extracted from the fresh leaves of the tea tree; it is rich in a variety of biological active substances, such as terpenes [15]. Because of its broad spectrum of antibacterial [16], anti-inflammatory, and other biological activities [17], it has been widely used in medicine, food, and other industries. However, plant EOs have some disadvantages, such as a poor water solubility and easy decomposition [18], which limit their further application. A nanoemulsion is an isotropic, transparent/translucent, heterogeneous system of two immiscible liquids, consisting of a fine dispersion of drugs in nanodroplets. It is stabilized by an interfacial layer of emulsifiers and co-emulsifiers. They are thermo-dynamically and kinetically stable systems, with an extremely small droplet size (20 to 400 nm), uniform size distribution, and different physicochemical and biological properties to those of other emulsions (>500 nm) [19]. NE preparation processes are divided into low-energy and high-energy methods. As an appropriate method for a small test benchmark, the preparation of NE via ultrasonic irradiations is an effective method of decreasing the mean particle size [20]. Some researchers prepared their EO NEs via the ultrasonic emulsification method. For example, Hatice Yazgan et al. found that lemon NE, prepared using ultrasonic emulsification, exhibits a stronger antimicrobial effect against *S. aureus* and *E. faecalis*, compared to both 100% and 10% lemon EO [21]. Ginger EO NE, prepared using zein and sodium caseinate as co-surfactants, exhibited a superior bactericidal activity against *S. aureus* and *P. aeruginosa*, compared to the bulk EO [22]. To enhance the properties of BB and TTO, they also can be encapsulated into NE. In generally, the oil phases commonly used in the preparation of NE in other studies include sopropyl myristate, castor oil, soybean oil, isopropyl palmitate, etc. Compared to these ordinary oils, plant EO can not only be used as an oil phase, but also shows multiple pharmacological activities, such as anti-inflammatory, antibacterial, and antioxidant activities; this means that the NE prepared from plant EO will have the characteristics of “medicine and pharmaceutical excipients all-in-one”.

The surfactant is the decisive factor in ensuring the stability of an NE, including synthetic surfactants and natural surfactants [23]. Studies have shown that polysorbate-80 and other synthetic surfactants have a slow toxicity, and a series of biosafety risks and side effects [24,25,26]. Natural surfactants mainly include polysaccharides, proteins, phospholipids, and saponins; they have the advantages of biodegradability, low toxicity, and environmental friendliness [27,28]. In addition, some of them have certain pharmacological activity; for example, ginseng saponins not only act as a natural surfactant to form and stabilize O/W emulsions, but also have anti-diabetes, anti-cancer, anti-fatigue, anti-ageing, and neuroprotective functions [29]. According to the idea of “medicine and pharmaceutical excipients all-in-one” in traditional Chinese medicine, it may be an effective way to find green and safe natural surfactants in traditional Chinese medicine, which can improve the solubility of drugs, and reduce the toxicity of excipients, at the same time. Glycyrrhizic acid is one of the important active components in *Glycyrrhiza uralensis Fisch*; it consists of one molecule of glucose, and one molecule of glycyrrhetinic acid [30]. It has multiple pharmacological activities, including anti-inflammatory, anti-oxidative, anti-viral, immunoregulatory, anti-cancer, and anti-diabetic functions [31], but also play the role of a natural stabilizer, and can form micelles in an aqueous solution, and play a solubilizing role for insoluble components [32].

**Figure 1:**
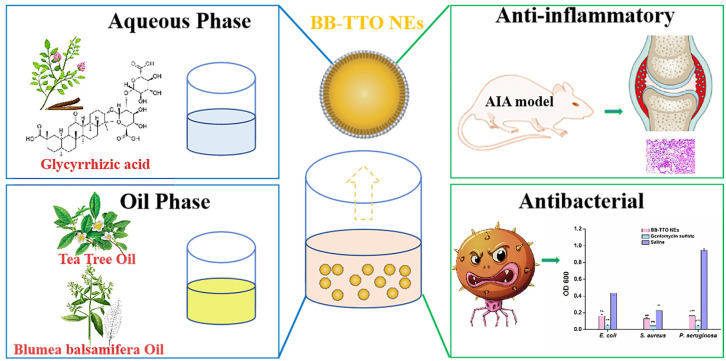
The preparation and biological activity evaluation of BB-TTO NEs (* *p* < 0.05, ** *p* < 0.01 and *** *p* < 0.001, as compared with saline).

In this study, glycyrrhizic acid was employed as a botanical surfactant, while BB and TTO constituted the mixed oil phase, to evaluate the feasibility of formulating a novel bioactive NE. The physicochemical properties of the obtained NEs were characterized, and their antibacterial and anti-inflammatory activity was also evaluated (Figure 1). This could improve the stability of plant EO, broaden its application range, and provide a new idea for the development of “medicine and pharmaceutical excipients all-in-one” bioactive NE and green drug delivery systems, instead of synthetic surfactants and common oil phases.

## 2. Results and Discussions

### 2.1. Single Factor Experiments to Optimize the Prescription of BB-TTO NEs

**Figure 2:**
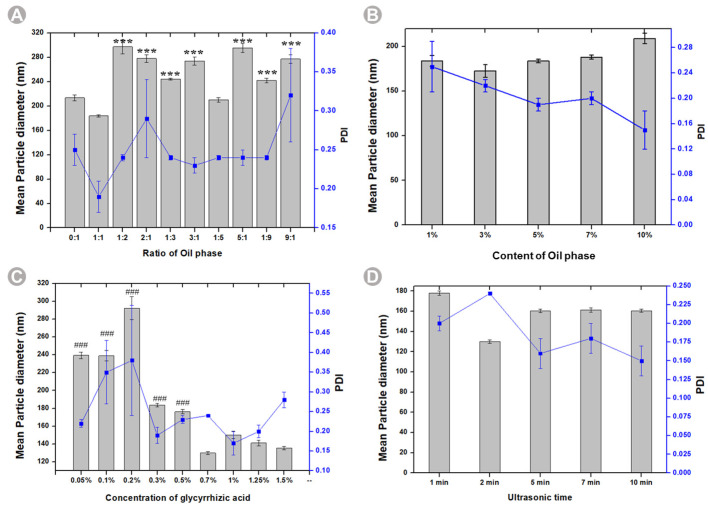
Prescription screening of BB-TTO NEs: (**A**) ratio of BB to TTO; (**B**) content of oil phase; (**C**) concentration of glycyrrhizic acid; (**D**) ultrasonic time. (*** *p* < 0.001, significantly different from the oil ratio 1:1 group; ### *p* < 0.001, significantly different from the 0.7% glycyrrhizic acid group).

We investigated the vital parameters, mean particle size, and Polydispersity Index (PDI) concerning the optimization of BB-TTO NE formulations. The oil type and content play an important role in determining their ability to form and stabilize emulsions [33]. Therefore, we evaluated the effect of the BB to TTO ratio and oil content on BB-TTO NEs. As expected, the ratio of BB to TTO exhibited a significant impact on the average particle size and stability of the NE. At a 1:1 ratio, the NE displayed an average particle size of 183.78 nm, while other proportions of BB and TTO resulted in a particle size exceeding 200 nm. Notably, the PDI was most stable at 0.20 ± 0.02 for the 1:1 ratio (Figure 2A and Figure S1). Regarding the oil phase composition, NEs containing a 1%, 3%, and 7% oil content demonstrated instability, characterized by phase separation and floating oil beads. Conversely, NEs containing a 5% and 10% oil phase content exhibited a homogenous milky white appearance, indicative of their stability and successful emulsification. Additionally, the NE at 5% oil phase content had a smaller particle size, and a relatively stable PDI. Based on appearance, average particle size, and PDI considerations, the optimal prescription for oil phase content was determined to be 5% (Figure 2B and Figure S2). A surfactant is necessary for facilitating the formation of, and improving the kinetic stability of, NEs. Research has demonstrated the critical role of an appropriate surfactant concentration in maintaining the stability of NEs [34]. Consequently, we investigated the effects of varying concentrations of the surfactant (glycyrrhizic acid). The results indicated that NEs prepared with 0.3%, 0.5%, and 0.7% glycyrrhizic acid exhibited a favorable appearance. However, NEs with a lower glycyrrhizic acid concentration (e.g., 0.05%, 0.10%, and 0.20%) showed the phenomenon of oil droplet suspension. This could be because the oil/water interface could not be stabilized with so few available surfactants [35]. On the contrary, when the concentration of glycyrrhizic acid exceeded 1%, precipitation was observed in all the NEs, and the precipitation amount increased with the increase in the concentration of glycyrrhizic acid. It is plausible that the high surfactant concentrations surpassed a particular threshold, at which a highly viscous liquid formed around the droplet phase, thus impeding the spontaneous breakup of the oil–water interface [36]. At 0.7% glycyrrhizic acid content, the mean particle size was 130.00 nm, and the PDI was 0.24, which led us to select this content as the optimal surfactant dosage (Figure 2C and Figure S3). Finally, we assessed the influence of ultrasonic time on the average particle size of the NE. NEs prepared with ultrasonic times of 1, 2, 5, 7, and 10 min displayed a uniformly milky appearance (Figure 2D and Figure S4). When the ultrasonic time was less than 5 min, the particle size or PDI was large. However, stability in terms of the average particle size and PDI was achieved when the ultrasonic time exceeded 5 min. Consequently, the optimal ultrasonic time for NE preparation was determined to be 5 min.

### 2.2. Characterization of BB-TTO NEs

The optimal formulation was determined through a single-factor experimental approach, wherein the ratio of the oil phase (BB: TTO) was set at 1:1, the oil phase content was fixed at 5%, the glycyrrhizic acid amount was established at 0.7%, and the ultrasonic time was maintained at 5 min for the preparation of BB-TTO NEs. Subsequently, a thorough characterization of their physicochemical attributes was conducted.

#### 2.2.1. Morphological Analysis

**Figure 3:**
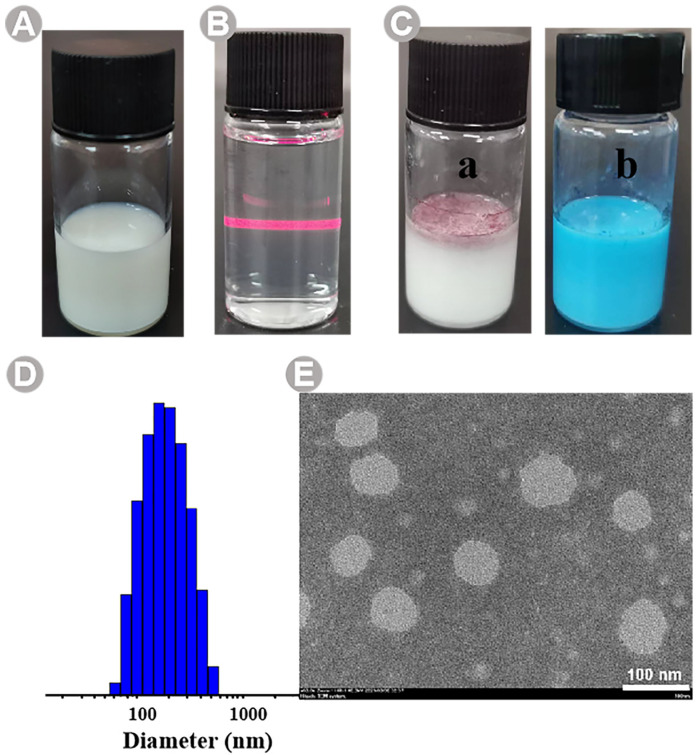
Physicochemical characterization of BB-TTO NEs: (**A**) appearance and shape; (**B**) Faraday–Tyndall effect after 1000-time dilution; (**C**) type identification ((a) Sudan red, (b) methylene blue); (**D**) particle size distribution diagram; (**E**) TEM photos.

It can be seen from Figure 3A that the prepared NE is uniform milky white, without stratification, precipitation, or suspension of oil droplets, etc., so it is judged that the appearance and shape of the NE is good. The Faraday–Tyndall effect is a phenomenon in which the particles in a colloid scatter the beams of light that are directed at them, and a path appears in the visible light, which can be used to judge the nanoscaled assembly and formation of nanoparticles [37]. After the dilution of the BB-TTO NEs, an obvious Faraday–Tyndall effect can be seen, confirming the presence of nanoparticles (Figure 3B). Then, the BB-TTO NEs are subjected to a TEM examination, to analyze the structure and morphology. It can be seen from the TEM image in Figure 3E that the droplets of the NE have a relatively good microscopic morphology, with a smooth, spherical structure, and a uniform size, without an obvious adhesion phenomenon.

#### 2.2.2. Mean Particle Size, PDI, and Zeta Potential

The mean particle size and PDI are the key parameters that affect the quality, homogeneity, and dispersibility of an NE, and can be determined via dynamic light scattering (DLS) [38]. The penetration of the drugs encapsulated in nanoemulsions is enhanced when the droplet size is less than 500 nm [39]. The average size for the BB-TTO NEs recorded is 160.32 ± 1.92 nm (Figure 3D), achieving the desired particle size, which is far less than 500 nm. PDI is the ratio of standard deviation to mean droplet size, which can reflect homogeneity in particle size in a solution; a PDI lower than 0.2 is desirable, as it implies that the NE droplets are almost similar in size, and free of adhesion and aggregation [38,40]. The PDI of BB-TTO NEs is 0.16 ± 0.02, which is an ideal value. The zeta potential can be used to evaluate the stability of particles in a suspension; if the absolute of the zeta potential value is greater than 30, the suspension can be considered stable [41]. The zeta potential average value of BB-TTO Nes is −50.94 ± 9.21 mV (Tables S1–S4), which can evade aggregation, and increase the stability of the emulsion. These results indicated that the emulsion prepared using this method has a good uniformity and repeatability.

#### 2.2.3. Type Identification

To predict the type of NE formed under given conditions, the interaction of various components making up the NE must be estimated. If the chief surfactant is water soluble, it favors O/W emulsification and, conversely, if the surfactant is oil soluble, it favors W/O emulsification [42]. The type of NE was determined using the oil-soluble dye Sudan Red, and the water-soluble dye methylene blue [43]. The results showed that the diffusion rate of methylene blue in the BB-TTO NEs was faster than that of Sudan red, which indicated that the NE is O/W type (Figure 3C).

#### 2.2.4. pH, Viscosity, and Turbidity

The incorporation of glycyrrhizic acid (as the surfactant) led to a reduced pH in the BB-TTO NEs, with a value of 3.29, thus satisfying the criterion for a weak acidity in skin preparations [44,45]. The viscosity is an important parameter of a drug carrier used through topical or transdermal application, which can influence stability and spreadability; a lower viscosity is conducive to the penetration of the preparation into the skin [46]. The average viscosity of BB-TTO NEs was calculated to be 55.07 ± 0.79 mPa·s, using the NDJ-9S rotational viscosimeter, meeting the requirements for local skin administration. The turbidity measurement is a simple and low-cost method of defining the stability of an emulsion [47]. The absorbance of the NE at 650 nm was measured using the enzyme marker [48], and the turbidity of BB-TTO NEs was 6.62 cm^−1^ after dilution.

### 2.3. Stability Studies

**Figure 4:**
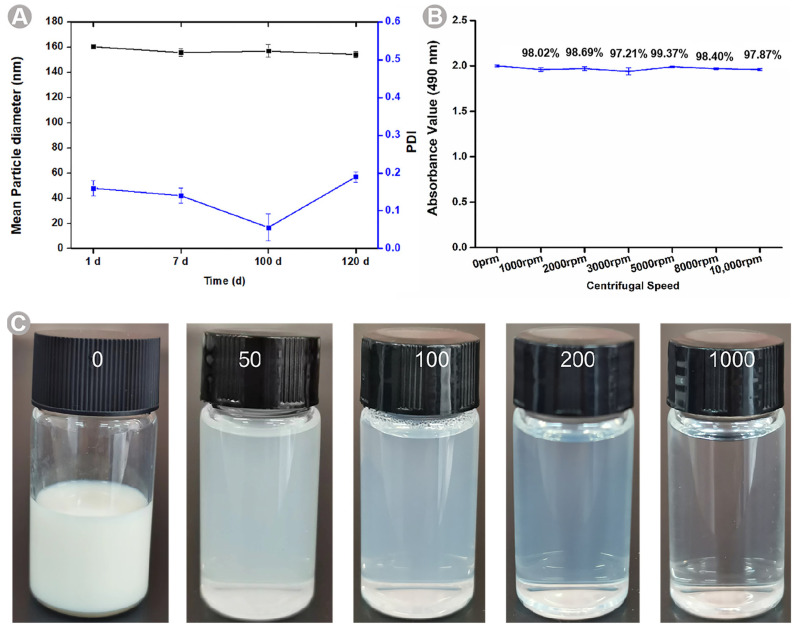
The stability studies of BB-TTO NEs: (**A**) stability of storage; (**B**) centrifugal stability; (**C**) stability of dilution.

The NE stability is an important factor, which explains the formulation’s shelf life. BB-TTO NEs were stored at room temperature for 7, 100, and 120 days. Remarkably, after the 120-day storage period, the NE displayed no signs of stratification, precipitation, demulsification, or any other unstable phenomena (Figure 4A and Table S5). NEs exhibit kinetic stability [49]. To assess the stability of BB-TTO NEs, a centrifugation method was employed to accelerate the emulsion breakage. Centrifugation was performed at the varying rotational speeds of 1000, 2000, 3000, 5000, 8000, and 10,000 rpm for 15 min. Remarkably, no phase separation or formation was observed throughout the experiments. Although the absorbance showed a decline with the increasing rotational speed, it remained within the range of 1.8–2.0. The values for the centrifugal stability coefficient of the BB-TTO NEs were over 97% (Figure 4B and Table S6), indicating that the prepared NEs had an excellent centrifugal stability. Then, the BB-TTO NEs were diluted 50, 100, 200, and 1000 times, as shown in Figure 4C; the phenomena of stratification and precipitation did not appear in the NE after different numbers of dilutions, indicating that BB-TTO NEs has a good dilution stability.

### 2.4. Cytotoxicity Studies of BB-TTO NEs

**Figure 5:**
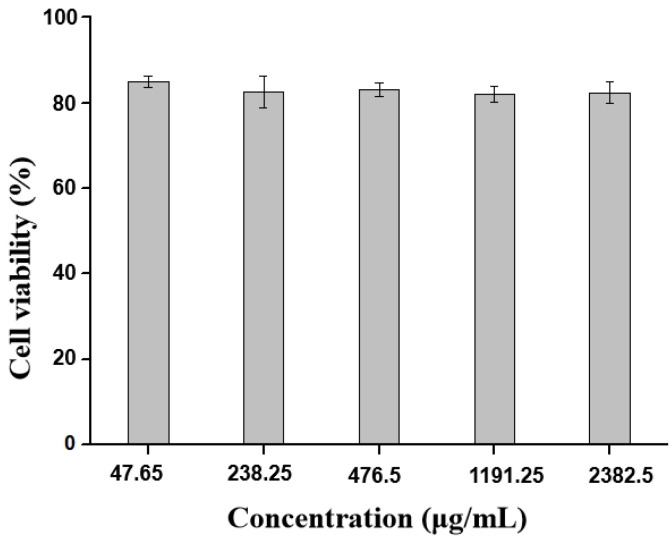
The cell viability of MC3T3-E1 cells cultured with increasing concentrations of BB-TTO NEs was recorded using an MTT assay. A cell treated with α-MEM only was set as a blank control. Data were presented as mean ± SD (*n* = 4).

The MTT assay is widely used to evaluate the in vitro cytotoxic effects of active drugs or biomaterials on cells, because the total mitochondrial activity corresponds with the percentage of viable cells in most cell populations [38]. Therefore, the in vitro cytotoxicity of the BB-TTO NEs was examined via MTT assay, using mouse preosteoblast MC3T3-E1 cells as the model cells. Figure 5 represents the impact of varying NE concentrations on the viability of cells. Among all the treatments with different concentrations of the conditioned media obtained from the BB-TTO NEs, no significant decrease in cell viability was observed, compared to the control cells. The results indicated that BB-TTO NEs exhibited a great biosafety with MC3T3-E1 cells, even up to 80% when the dosage ranged between 47.65 and 2382.5 μg/mL. Tatiiana Zanela da Silva Marques et al. [50] found that an NE containing propranolol is nontoxic, and maintains a growth of over 80% in the fibroblast cell. Similar findings from Niharika Walia et al. [51] also showed that an NE prepared by combing pea protein and tween 80 could keep the viability of Caco-2 cells above 80%.

### 2.5. Antibacterial Evaluation

**Figure 6:**
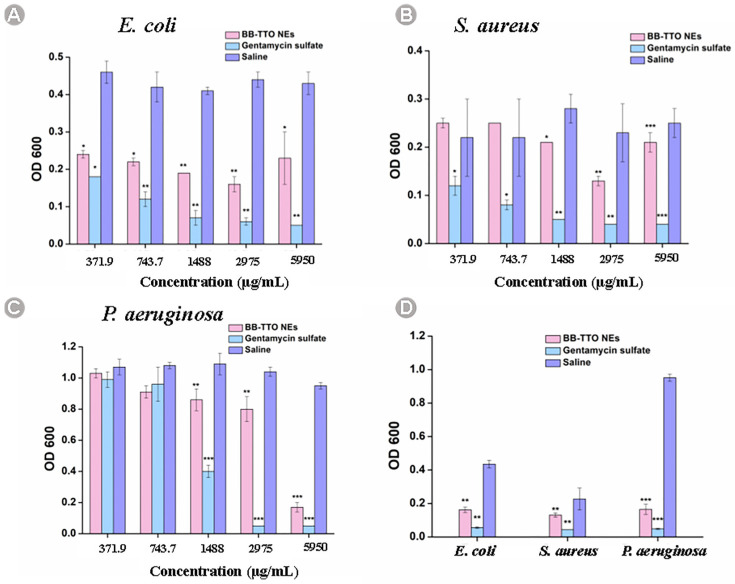
Evaluation of the inhibitory concentration of BB-TTO NEs on different bacteria. (**A**) *E. coli*; (**B**) *S. aureus*; (**C**). *P. aeruginosa*; (**D**) the absorbance values of the MIC of BB-TTO NEs, gentamycin sulfate, and saline for different bacteria (* *p* < 0.05, ** *p* < 0.01 and *** *p* < 0.001, as compared with saline).

The minimum inhibitory concentration (MIC) test was employed to provide insight into the quantitative measurement of antibacterial activity [52,53]. Figure 6 and Table 1 listed the absorbance values of BB-TTO NEs at different concentrations and MICs; compared with the normal saline group, BB-TTO NEs can significantly inhibit the activity of bacteria in a concentration-dependent manner. The MIC of BB-TTO NEs against *E. coli* and *S. aureus* was determined to be 2975 μg/mL, while the MIC against *P. aeruginosa* was found to be 5950 μg/mL. Currently, the underlying cause behind the elevated OD values of *E. coli* and *S. aureus* at the concentration of 5950 µg/mL of BB-TTO NEs remains uncertain. BB, TTO, and glycyrrhizic acid, employed in this investigation, all possess inherent antibacterial properties. Investigating whether these three components interact synergistically, or if there is an optimal antibacterial concentration, is of paramount importance in elucidating the antibacterial effects and potential mechanisms of BB-TTO NEs. These aspects require further systematic exploration through experimental studies in future research endeavors. The BPEO NE prepared by Nie et al. [54] using black pepper EO and Tween 80 showed an MIC against *E. coli* and *S. aureus* of 12.5 mg/mL. In contrast, the FLO NE prepared by Azmi et al., [38] using Fish by-product oil, lemon oil, and Tween 80, exhibited MIC values of 250 mg/mL and 62.5 mg/mL against *E. coli* and *S. aureus*, respectively. Comparatively, BB-TTO NEs demonstrated a superior antibacterial effect against these bacteria, compared to the other EO NEs, highlighting their potential in the field of antibacterial applications.

In a general way, the antibacterial activity of the NE is dependent on its composition, and experimental strains. It has been reported that BB, TTO, and glycyrrhizic acid have an excellent bacteriostatic effect on *S. aureus* [12,16,55]. Yang et al. [12] proved that BB can destroy the permeability of the cell membrane, and inhibit the synthesis of bacterial nucleic acid and protein against *S. aureus*. Li et al. [56] reported that TTO may exert its antimicrobial effects by compromising the cell membrane, resulting in the loss of the cytoplasm, and organelle damage, which ultimate leads to cell death. Mohammed et al. [57] reported that glycyrrhizic acid exerts antibacterial effects by affecting the permeability of the bacterial cell membrane, biofilm formation, and efflux activity. When used together, TTO, BB, and glycyrrhizic acid can jointly interfere with bacterial cell membranes, and are expected to play a highly effective antibacterial role. In addition, after the BB, TTO, and glycyrrhizic acid are prepared and added to the NE, the small lipid particles inside the NE are capable of transporting them towards the surface of the bacterial cell membranes, and enhancing antibacterial activity [58]. Although the mechanism of the antibacterial activity of BB-TTO Nes remains unknown, the dominating target is the cell membrane of the strain, as it influences the basic processes inside, and at the membrane, inhibits enzyme activity, prevents the absorption of nutrients, and even lytic cells, all of which might lead to growth inhibition or death in bacteria [59]. The specific antibacterial mechanism needs to be further explored in future work.

### 2.6. Anti-Inflammatory Activity

**Figure 7:**
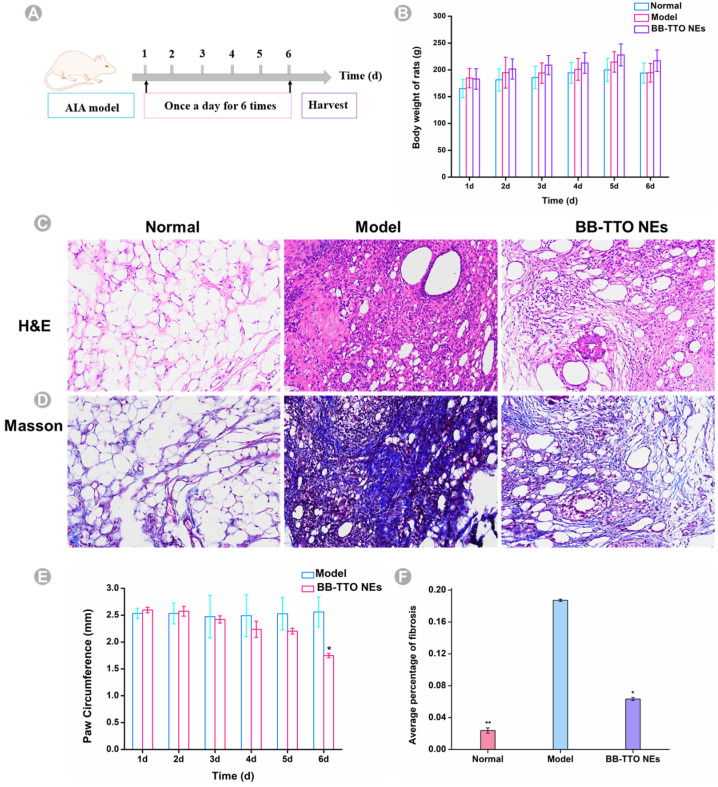
The anti-inflammatory activity of BB-TTO NEs was analyzed in an AIA rat model. (**A**) Experimental strategy for the therapeutic potential study of BB-TTO NEs; (**B**) body weight of AIA rats with or without treatment; (**C**) H&E staining images of synovial tissues with or without treatment; (**D**) Masson staining of synovial tissues with or without treatment; (**E**) paw swelling thickness in AIA rats with or without treatment; (**F**) compared with the model group, the average percentage of fibrosis before and after treatment (* *p* < 0.05, ** *p* < 0.01, as compared with model group).

Notably, both BB, TTO, and glycyrrhizinic acid have been reported to have anti-inflammatory effects [13,17,60]. Consequently, we investigated the therapeutic potential of BB-TTO NEs in an AIA rat model, to evaluate their anti-inflammatory activity. After the complete Freund’s adjuvant induced arthritis SD rat model was conducted, we treated rats once a day with 11,900 μg/mL BB-TTO Nes, according to the timeline shown in Figure 7A; the paw swelling was monitored every day. First, we recorded the body change in the rats, to evaluate the life quality. There was no significant change in the weight of the rats in the different groups (Figure 7B). As shown in Figure 7E, the AIA rats without treatment maintained a high paw thickness, because of the joint inflammation [61]; in the BB-TTO NEs group, the swelling symptoms were gradually decreasing, especially on the sixth day (*p* < 0.05). The rats were sacrificed on day 6 after their treatment with BB-TTO Nes, and subjected to a histopathological examination. The results of the H and E staining in Figure 7C show an intense inflammatory cell infiltration, consisting of macrophages, neutrophils, etc., synovial thickening, pannus formation (the infiltration of adaptive immune cells into the synovial sublining form a hallmark “pannus” formation at cartilage–bone interfaces) [62], and cartilage erosion were observed in the ankle joints of the model group rats. The joint structure among the blank group was normal; no pannus formation or inflammatory cell infiltration was observed. Compared with the model group, the synovial cell proliferation, pannus formation, and inflammatory cell infiltration were significantly improved in the NE group. Some investigators have also demonstrated the anti-inflammatory activity of EO NE using models of arthritis. Li et al. [63] prepared a sanse powder EO NE (SP-NE) and also found less inflammatory cell infiltration in the synovial tissue of osteoarthritis rats treated with SP-NEs. Similar results were reported by Mohammadifar et al. [64]. They indicated that NEs containing peppermint and rosemary EO reduced osteoarthritis pain, via increasing the antioxidant capacity, and improving the histopathological features of the rats’ knee joints. Masson staining also showed that the average percentage of fibrosis after BB-TTO NE treatment was significantly lower than that among the model group; it was only 0.06%, while the amount in the model group was about three times that of the BB-TTO NE group (Figure 7D,F). The above results demonstrate that BB-TTO NEs perform the biological activity of inhibiting inflammation in specific arthritis sites.

Studies have shown that glycyrrhizic acid can play an anti-inflammatory role, by selectively blocking the triggering of phosphorylation by inflammatory mediators (mainly enzymes), and blocking or neutralizing the production of pro-inflammatory chemokines, such as IL-8 and eotaxin 1 [65]. TTO has been shown to exhibit anti-inflammatory properties, by preventing NF-κB and inhibitor of NF-κB (IκB) phosphorylation, which is important for NF-κB activation and the expression of inflammatory cytokines, including TNF-α and IL-6 [66]. The anti-inflammatory activity of BB works through inhibiting TLR4/NF-kB signaling pathways and NLRP3 inflammasome activation [67]. NEs possess various advantages that increase their ability to enhance skin permeation, such as nanosized droplets and a lowered interfacial tension [68]. Therefore, the BB-TTO NEs in this study may penetrate the skin of AIA rats, and have a synergistic anti-inflammatory effect by inhibiting the activation of inflammatory signaling pathways, and the release of inflammatory factors. If it is used as a nanodelivery platform for arthritis treatment drugs in the future, it can offer the full advantage of the combined effect of drugs and pharmaceutical excipients, and play a synergistic role in the treatment of arthritis.

## 3. Materials and Method

### 3.1. Materials

BB was provided by Guizhou AiYuan Ecological Medicine Development Ltd. (Guizhou, China); TTO was purchased from Aladdin (Shanghai, China); Gentamycin sulfate and the bacterial growth media including LB Broth were obtained from Solarbio (Beijing, China). Complete Freund’s Adjuvant (CFA) was purchased from Sigma (St. Louis, MO, USA). *P. aeruginosa*,* E. coli*, and *S. aureus* were contributed by Guizhou Medical University. SD rats were provided by Guizhou University of Traditional Chinese Medicine.

### 3.2. Preparation of BB-TTO NEs

BB-TTO NEs were prepared via ultrasonic emulsification, with a slight modification [69]. The oil phase consisted of TTO and BB, whereas the aqueous phase was comprised of distilled water and glycyrrhizic acid (surfactant). After the preparation of the two phases, the aqueous phase was added to the oil phase dropwise, and stirred gently with a magnetic stirrer at room temperature for 15 min. Then, the coarse emulsion was subjected to a JY92-IIN ultrasonic homogenizer (Ningbo Scientz Biotechnology Co., Ltd., Ningbo, China) under the following homogenizing conditions: duration, 2 min with a 5-s pulse turned on and a 5-s pulse turned off; power, 160 W; and ultrasonic frequency, 20 kHz.

### 3.3. Single-Factor Experiments to Optimize the Prescription of BB-TTO NEs

The effects of parameters on the mean particle size and PDI of BB-TTO NEs were systemically investigated using single-factor experiments, including the ratio of TTO and BB (0:1, 1:1, 1:2, 2:1, 1:3, 3:1, 1:5, 5:1, 1:9, and 9:1), the content of oil (1%, 3%, 5%, 7%, 10%), the concentration of glycyrrhizic acid (0.05%, 0.1%, 0.2%, 0.3%, 0.5%, 0.7%, 1%, 1.25%, 1.5%) and the ultrasonic time (1, 2, 5, 7, and 10 min). The best prescription of BB-TTO NEs was selected by observing the appearance, and measuring the particle size and PDI of the NEs.

### 3.4. Characterization of Physicochemical Properties of BB-TTO NEs

The nanoproperty of BB-TTO NEs was represented by a Faraday–Tyndall effect, the particle size, PDI, and zeta potential value of BB-TTO NEs were analyzed using the dynamic light scattering instrument’s measurement chamber (Beckman nanoscale particle size analyzer DelsaMax PRO, Beckman Coulter, Inc, Bria, California, USA). All samples were diluted using distilled water at a ratio of 1:40; each measurement was repeated at least 3 times to ensure the repeatability of the analysis.

The type of BB-TTO NE was determined according to the diffusion rate of the oil-soluble dye Sudan red (red) and water-soluble dye methylene blue (blue). If the diffusion rate of red is faster than that of blue, it is W/O type NE [43].

The morphology of the BB-TTO NEs was analyzed via transmission electron microscope (TEM, Hitachi, Tokyo, Japan). The sample was diluted in water at 1:40; 200 μL of the diluted BB-TTO NEs was dropped on the surface of the copper grid, and the excess liquid was removed with filter paper. The sample was stained with 1% phosphotungstic acid solution (pH 7.0), and then the material deposited on the grid was analyzed via TEM.

The pH of the BB-TTO NEs was analyzed using a digital pH meter (PHS-3E, Shanghai Yi Electrical Scientific Instruments Co., LTD, Shanghai, China) at room temperature, and the viscosity of BB-TTO NEs was measured using an NDJ-8S rotary viscometer (rotor 0, speed 6.0 rpm/min).

The prepared NE was diluted 50 times with deionized water, and then the turbidity was measured using an enzyme marker at the wavelength of 650 nm. The turbidity was repeated three times, and the average value was calculated according to the formula [70]:

T = 2.303A × D/L(1)

T is the turbidity, A is the absorbance at wavelength 650 nm, D is the dilution ratio of NE, L is the sample path depth.

### 3.5. Stability Studies

To evaluate the storage stability of BB-TTO NEs, visible observations and physical analyses of the size and PDI changes were carried out over 120 days of storage at room temperature [71]. The centrifugation of the NE ensured kinetic stability when studied via creaming, sedimentation, coalescence, and phase separation [19]. For 15 min, 2 mL BB-TTO NEs were centrifugated at 1000, 2000, 3000, 4000, 5000, 8000, and 10,000 rpm, respectively. The appearance properties before and after centrifugation were observed, and the absorbance changes were measured at 490 nm, using a thermo scientific^TM^ Multiskan sky, and the centrifugal stability coefficient K was calculated using the following formula [70]:

K(%) = A_1_/A_0_ × 100% (2)

A_0_: the absorbance value of BB-TTO NEs at 490 nm wavelength before centrifugation, A_1_: the absorbance value of BB-TTO NEs at 490 nm wavelength after centrifugation.

For dilution stability, BB-TTO NEs were diluted using distilled water at a ratio of 1:50, 1:100, 1:200, and 1:1000, respectively, and the appearance of NEs was observed before and after dilution.

### 3.6. Cytotoxicity Studies of BB-TTO NEs

An MTT assay was used to evaluate the cytotoxicity of BB-TTO NEs. MC3T3-E1 cells were seeded in a 96-well plate (5000 cells per well), and were incubated for 24 h at 37 °C with 5% CO_2_. Subsequently, the treatment plane was added, with different doses of BB-TTO NEs (47.65, 238.25, 476.5, 1191.25, and 2382.5 μg/mL), compared with nontreated cells. After another 24 h of incubation, the cells were washed using PBS at least 3 times. A 10% MTT reagent was added into each well, following the standard method. Finally, a microplate spectrophotometer was used to measure the absorbance of formazan at 570 nm. The cell viability was estimated using the following equation:

Cell viability (%) = (OD_sample_/OD_control_ × 100%) (3)

### 3.7. Antibacterial Evaluation

The antibacterial activity of BB-TTO NEs was assessed using the widely recognized MIC method, as described in the literature [52]. To execute this, the BB-TTO NEs were assessed via a broth micro-dilution assay, using a sequential dilution in sterile 96-well microplates. Initially, 100 μL sterilized LB Broth was added to each of the rows. Wells 1–11 were augmented with 100 μL of BB-TTO Nes, and diluted serially, to form a sequence of concentrations from 5950 μg/mL to 371.9 μg/mL. The final well served as the growth control. The microplates were incubated for 24 h at 37 ℃. Each assay was replicated in triplicate, and the absorbance was measured at 600 nm. The MIC value was taken at the lowest concentration of antibacterial agents that inhibits the growth of bacteria (minimum OD_600_ value). The positive and negative controls used were gentamycin sulfate at 50 μg/mL, and saline, respectively.

### 3.8. Anti-Inflammatory Activity

The AIA model was established according to the previous study, and slightly modified [72]. All animal studies were performed in accordance with the protocols approved by the Laboratory Animal Welfare and Ethics Review Committee of Guizhou University of Traditional Chinese Medicine (No. GZY20220112). Briefly, twelve SD rats were selected, and each rat was injected intradermally into the right posterior plantar with 0.1 mL complete Freund’s adjuvant, to induce inflammation, and 100 μL CFA was injected again on the third day, to create early-stage arthritis SD rats. After 7 days, the rats were randomly divided into three groups: the normal group, model group, and treatment group. The treatment and model groups were coated with BB-TTO NEs and saline, respectively, and their weight and paw thickness were recorded every day. After 6 days of treatment, the rats were sacrificed, and the synovial tissue was collected for hematoxylin–eosin (H and E, a classic standard tissue section staining method widely used in the inspection of tissue components for pathological analysis that is applicable in all organs and disease models) and Masson staining. IPP6.0 software was used to measure the fibrosis area in the Masson staining photographs; the average fibrosis proportion represented the fibrosis level of the sample, and the calculation formula is as follows:

Fibrosis proportion = Fibrosis area/Tissue area × 100%(4)

### 3.9. Statistical Analysis

All results were measured at least three times, and expressed as mean ± standard deviation. Student’s *t*-test or one-way analysis of variance (ANOVA) was used to assess the differences among groups. When *p* < 0.05, the difference was considered statistically significant, * *p* < 0.05, ** *p* < 0.01, *** *p* < 0.001.

## 4. Conclusions

In this study, a new type of nanoemulsion was prepared, with glycyrrhizic acid as the surfactant, and BB and TTO as the oil phase. The nanoemulsion was O/W, regularly spherical, and the size was about 160 nm. BB-TTO NEs had inhibitory effects on *E. coli*, *S. aureus*, and *P. aeruginosa*. More interestingly, compared with the control group, after 6 days of BB-TTO NE application, the joint-swelling degree of arthritis model rats was reduced, the number of inflammatory cells in the synovial tissue was significantly reduced, the symptoms of connective tissue hyperplasia were significantly relieved, and the average percentage of fibers was significantly reduced. This experiment confirmed the feasibility of using glycyrrhizic acid as a natural surfactant to prepare new green nanopreparations, and provided a new choice and direction for the development of new green nanopreparations. At the same time, it also provided a basis and reference for the application of EOs in cosmetics and other fields. However, the nanoemulsion’s potential anti-inflammatory and antibacterial mechanisms remain to be further studied.

## Figures and Tables

**Table 1:** MIC of BB-TTO NEs against different strains.

Strain	MIC (μg/mL)
*E. coli*	2975
*S*. *aureus*	2975
*P.* *aeruginosa*	5950

## Data Availability

The original contributions presented in the study are included in the article, and further inquiries can be directed to the corresponding author.

## Supplementary Materials

The following supporting information can be found online https://www.mdpi.com/article/10.3390/molecules28155889/s1: Figure S1: Effect of oil phase ratios on BB-TTO NEs; Figure S2: Effect of oil phase content on BB-TTO NEs; Figure S3: Effect of glycyrrhizic acid concentration on BB-TTO NEs; Figure S4: Effect of ultrasound time on BB-TTO NEs; Table S1: Zeta potential of NEs prepared with different oil phase ratios; Table S2: Zeta potential of NEs prepared with different oil phase contents; Table S3: Zeta potential of NEs prepared with different glycyrrhizic acid concentrations; Table S4: Zeta potential of NEs prepared with different ultrasound times; Table S5: Storage stability of BB-TTO NEs; Table S6: Centrifugal stability of BB-TTO NEs.
