# Supplementary material for: Fabrication and Biological Activities of All-in-One Composite Nanoemulsion Based on Blumea balsamifera Oil-Tea Tree Oil
Source: Molecules. 2023 Aug 5;28(15):5889. doi: 10.3390/molecules28155889 (PMC10420664; doi:10.3390/molecules28155889)
Supplement: Supplementary file 1 [file molecules-28-05889-s001.zip › molecules-2518486-supplementary.pdf]

Supplementary Materials

Fabrication and biological activities of all in one composite nanoemulsion based on Blumea balsamifera oil-Tea tree oil

Yue Zhu [1,2], Teng Chen [1,2], Tingting Feng [1], Jiaojiao Zhang [3], Zejing Meng [1], Ning Zhang[4], Gang Luo [5], Zuhua Wang [1,2]*, Yuxin Pang [1]*, Ying Zhou [1]*

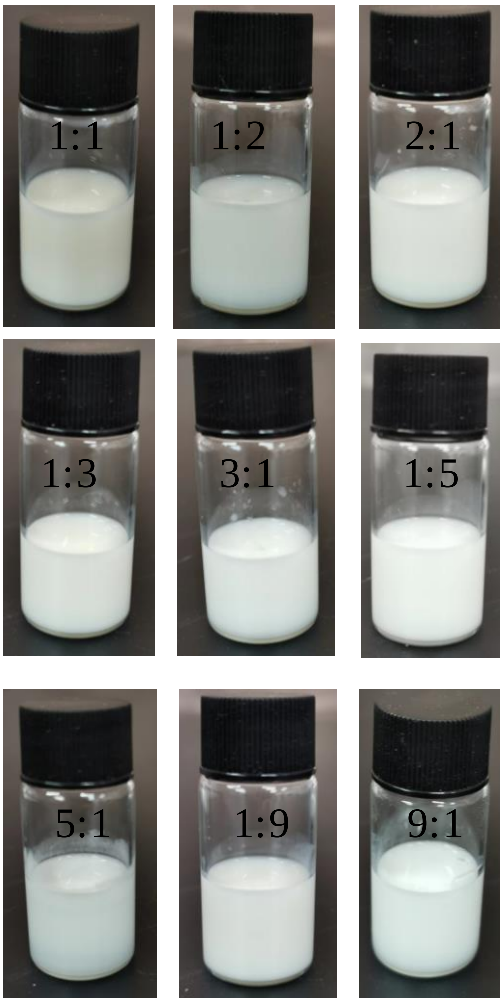

Figure S1. Effect of oil phase ratio on BB-TTO NEs.

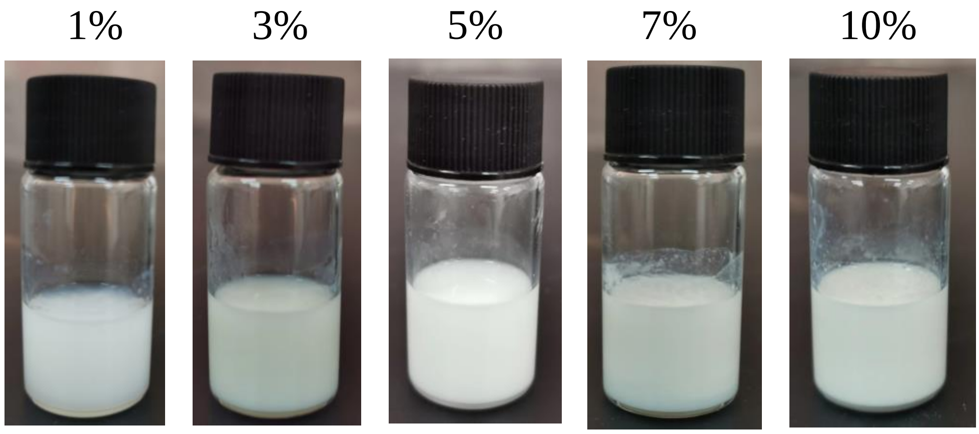

Figure S2. Effect of oil phase content on BB-TTO NEs.

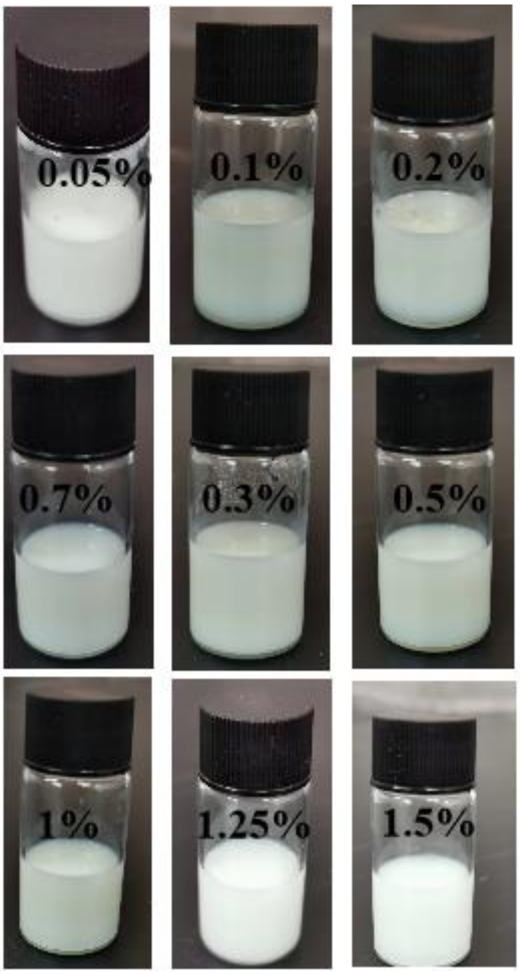

Figure S3. Effect of glycyrrhizic acid concentration on BB-TTO NEs.

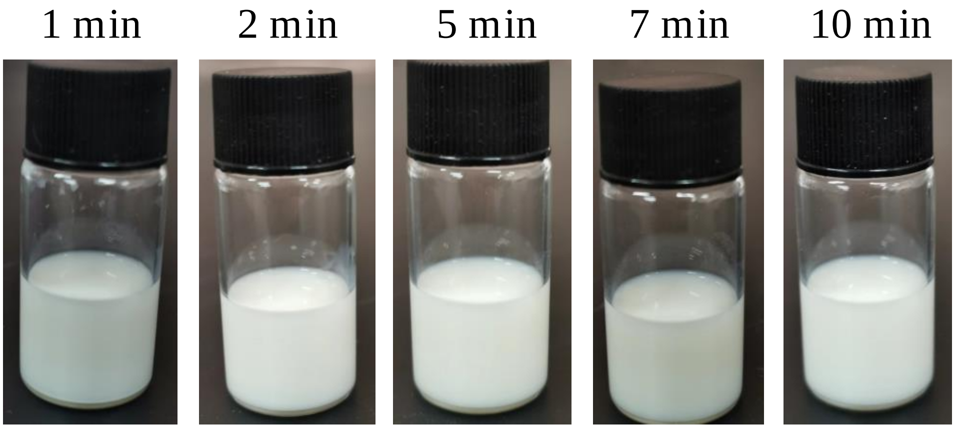

Figure S4. Effect of ultrasound time on BB-TTO NEs.

Table S1. Zeta potential of nanoemulsions prepared with different oil phase ratio.

Oil phase ratio	Zeta potential (mV)
1：1	-41.38 ± 1.05
1：2	-36.07 ± 0.75
2：1	-49.76 ±9.85
1：3	-43.61 ±0.82
3：1	-45.66 ±1.55
1：5	-38.86 ±1.70
5：1	-32.03±0.75
1：9	-41.01 ±0.93
9：1	-39.31 ±1.72

Table S2. Zeta potential of nanoemulsions prepared with different oil phase content.

Oil phase concentration (%)	Zeta potential (mV)
1	-21.19 ± 4.02
3	-41.29 ± 1.14
5	-38.50 ±1.19
7	-41.29 ±1.14
10	-47.49 ±5.15

Table S3. Zeta potential of nanoemulsions prepared with different glycyrrhizic acid concentrations.

Glycyrrhizic acid concentration (%)	Zeta potential (mV)
1：1	-41.38 ± 1.05
1：2	-36.07 ± 0.75
2：1	-49.76 ±9.85
1：3	-43.61 ±0.82
3：1	-45.66 ±1.55
1：5	-38.86 ±1.70
5：1	-32.03±0.75
1：9	-41.01 ±0.93
9：1	-39.31 ±1.72

Table S4. Zeta potential of nanoemulsions prepared with different utrasound time.

Ultrasound time (min)	Zeta potential (mV)
1	-25.99 ± 0.90
3	-36.37 ± 4.82
5	-50.94 ±9.21
7	-53.94 ±3.18
10	-55.18 ±4.13

Table S5. Storage stability of BB-TTO NEs.

	1 d	7 d	100 d	120 d
Size	160.32±1.8	155.6875±2.76	156.87±4.98	154.11±2.56
PDI	0.16±0.02	0.14±0.02	0.056±0.036	0.19±0.014

Table S6. Centrifugal stability of BB-TTO NEs.

centrifugal speed (rpm)	Phase Separation	Creaming	Flocculation	centrifugal stability coefficient K (%)
1000	Not detected	Not detected	Not detected	98.02
2000	Not detected	Not detected	Not detected	98.69
3000	Not detected	Not detected	Not detected	97.21
4000	Not detected	Not detected	Not detected	100.76
5000	Not detected	Not detected	Not detected	99.37
8000	Not detected	Not detected	Not detected	98.40
1000	Not detected	Not detected	Not detected	97.87
